# Diagnostic accuracy and clinical utility of the PHQ-2 and GAD-2: a comparison with long-format measures for depression and anxiety

**DOI:** 10.3389/fpsyg.2024.1259997

**Published:** 2024-05-10

**Authors:** Jón Ingi Hlynsson, Per Carlbring

**Affiliations:** ^1^Department of Psychology, University of Iceland, Reykjavík, Iceland; ^2^Department of Psychology, Stockholm University, Stockholm, Sweden

**Keywords:** generalized anxiety disorder questionnaire, patient health questionnaire, psychometric evaluation, internet-based psychotherapy, receiver operating characteristic analysis, item-option characteristic analysis

## Abstract

**Background:**

Anxiety and depression are highly prevalent and often comorbid mental disorders that are encompassed within the broad category of emotional disorders. The frequent comorbidity of anxiety and depression can pose challenges for accurate diagnosis and treatment which, in turn, highlights the need for reliable measurements that are simultaneously responsive to change and prevent non-response bias. Brief measures of anxiety and depression can potentially increase response rates due to their brevity and ease of administration. This study evaluates the psychometric characteristics, discriminative accuracy, and sensitivity to change of the Generalized Anxiety Disorder 2-item scale (GAD-2) and the Patient Health Questionnaire 2-item scale (PHQ-2) within a clinical population.

**Method:**

The sample comprised treatment-seeking participants (*n* = 3,411), screened (*n* = 2,477) to receive an internet-based psychotherapeutic intervention (cognitive-behavioral, psychodynamic, or waitlist).

**Results:**

Brief measures can effectively detect individuals who may be eligible for a diagnosis of depression and anxiety, not only prior to but also during and following the completion of psychological treatment. The discriminative ability of the GAD-2 was significantly greater during active treatment and at post-assessment compared with pre-treatment screening, although no such differences were found for the PHQ-2. Finally, endorsing the most severe response option on the GAD-2 and PHQ-2 was associated with a high probability of presenting with clinically relevant anxiety and depressive symptoms.

**Conclusion:**

Brief measures of anxiety and depression are viable instruments to screen for and monitor anxiety and depressive symptoms.

**Clinical trial registration:**

ClinicalTrials.gov, identifier NCT05016843.

## 1 Introduction

Anxiety and depression are highly prevalent mental disorders. The 12-month prevalence rates of anxiety and major depression is estimated at 12.7 and 7%, respectively (American Psychiatric Association, 2022; Szuhany and Simon, 2022) Recent global prevalence estimates indicate that 301.4 million people suffer from anxiety disorders and 279.6 million from depressive disorders (GBD 2019 Mental Disorders Collaborators, 2022). In the general population, around 17.2% of adults experience clinically significant depression or anxiety (Johansson et al., 2013), with comorbid presentations being the rule rather than the exception. Indeed, approximately 45.7% of individuals with lifetime major depression also have a lifetime history of one or more anxiety disorder (Kalin, 2020). Moreover, individuals seeking treatment for depression frequently experience anxiety symptoms and *vice-versa* (Choi et al., 2020). Taken together, anxiety and depressive disorders are the most common mental disorders (*cf.* GBD 2019 Mental Disorders Collaborators, 2022), but effective treatment is too often hindered by imprecise conceptualization and measurement of these disorders (*cf.* Watt, 2023; see also Fried et al., 2022).

Anxiety and depressive disorders are encompassed within the broad category of *emotional disorders*. Emotional disorders are characterized by frequent experiences of negative emotions coupled with maladaptive reactions to, and regulation of, such experiences which, in turn, increase the probability of future negative emotions and maintain the presenting disorder symptomology (*cf.* negative feedback loop; Bullis et al., 2019). These conditions are often undertreated and associated with lower quality of life, highlighting the need for further efforts regarding preventive and treatment interventions (Johansson et al., 2013).

Recently, researchers and clinicians (e.g., DeYoung, 2015; Sauer-Zavala and Barlow, 2021) have increasingly associated the core features of emotional disorders with the higher-order personality trait *neuroticism*. For instance, Sauer-Zavala and Barlow (2021) associate high levels of neuroticism with an increased propensity for negative emotional experiences, greater proneness to finding such experiences aversive, and, in turn, engagement in behavioral escape or avoidance strategies (*cf.* Bullis et al., 2019). Furthermore, neuroticism has garnered the greatest amount of neuroscientific research (*cf.* DeYoung et al., 2021) as it emerges as a robust predictor for psychopathology and mental disorder comorbidity (e.g., depression, anxiety, personality disorders; Lahey, 2009). As such, neuroticism is conceived of as a risk factor for psychopathological development (Lahey, 2009).

Neuroticism is closely associated with Jeffery Gray’s behavioral inhibition system (BIS); a system that reconciles conflicting goals (e.g., approach-avoidance) by recursively looping anxiety provoking content so as to increase its negative perceptual valence, which in turn facilitates behavioral resolution in favor of either approach or avoidance (Gray and McNaughton, 2000). Traits related to anxiety and depression are associated with BIS, which in turn implicates a disposition for passive avoidance (i.e., a tendency to avoid potential punishment and/or error by slowing or inhibiting behavior), and thus converge with neuroticism (DeYoung et al., 2021; see also Pickering and Corr, 2008). The BIS can be conceived of as the inverse of Panksepp’s (1998) SEEKING system. Sustained activation of the SEEKING system encourages exploratory behavior through sustained attention. However, an interruption of SEEKING system activation (e.g., due to threats or use of neuroleptics) can disrupt exploratory behaviors and motivation, and thus inversely predict depression (Panksepp and Watt, 2011; Davis and Montag, 2019; for a review, see Watt, 2023).

It is imperative for researchers to be aware of some of the faults associated with the key concepts underpinning their work when devising clinical scales. Although a deep conceptual analysis of anxiety and depression is beyond the scope of this paper, a significant issue in the DSM diagnostic classification system’s definition of major depression needs to be addressed. The main symptoms of depression are identified by the presence of “depressed mood” and/or “anhedonia” (American Psychiatric Association, 2022). However, the DSM does not provide a comprehensive description of what a depressed mood entails. This omission results in a circular situation where depression is identified by the presence of depression itself. Similarly, the DSM defines anxiety disorders as “disorders that share features of excessive fear and anxiety” (American Psychiatric Association, 2022, p. 215). In colloquial terms, this means that anxiety is defined by experiencing anxiety. This creates a conceptual circular reasoning problem that behavioral psychologists have attempted to highlight (Friman and Dymond, 2020).

The conceptual issues of circular reasoning evident in the DSM have not received the academic discussion they deserve. This oversight may be a contributing factor to the stagnation of theoretical understanding and treatment advancement for major depression (Watt, 2023). For instance, Watt and Panksepp (2009) argue against the vague definition of the depressed mood state, stressing the need to differentiate major depression from simple sadness (see also Wakefield et al., 2017). They suggest that the core psychopathology depression may be cojoined in a diminished sense of hope (*cf.* “depressed mood”), and a lack of motivation to seek or enjoy rewards (*cf.* “anhedonia”). However, they emphasize that these aspects of depression are distinctly different from simple sadness. Individuals with depression have a tendency to give up easily when faced with challenges, anticipate defeat or failure, and have pervasive pessimistic thoughts across various life domains (for a review, see Watt, 2023). Moreover, 10,377 unique symptom profiles of depression have been identified (Fried et al., 2020). This further suggests that current models of depression may not possess sufficient explanatory power to guide researchers in furthering our theoretical understanding of major depressive disorder. When all of this is juxtaposed with high comorbidity rates between depressive and anxious disorders, it becomes evident that more research is needed to fully understand the underlying mechanisms of emotional disorders in general, dovetailing with the abovementioned findings that suggest that emotional disorders, such as depression and anxiety, collapse under the single higher-order factor of neuroticism (DeYoung, 2015; Sauer-Zavala and Barlow, 2021).

Relatedly, much disagreement is evident in the clinical psychopathology literature regarding comorbidity of anxiety and depressive disorders in general and the diagnostic specificity of generalized anxiety specifically (e.g., Nemeroff, 2020; Roemer and Orsillo, 2020). For instance, Nemeroff (2020) raises the point that general anxiety may be the *forme fruste* (i.e., disguised precursor) for major depression; a claim that aligns with the higher-order dimensional trait diathesis (i.e., neuroticism) discussion above (for a detailed review on anxiety and depression and their relation to separation distress, see Watt, 2023). However, although depression and anxiety share common (non-specific) features, they are not identical emotional states. For instance, Beck (1976) proposed that depression and anxiety could best be differentiated by their cognitive content, wherein depressed individuals are increasingly prone to self-deprecating thoughts while anxious individuals increasingly fixate on potential dangers. Negative beliefs about oneself, the world, and the future stem from self-deprecatory thoughts in depression, while an excessive focus on potential dangers leads to the amplification of threats, their perceived probability, and potential harm in anxiety (*cf.* differential temporal orientation; Eysenck and Fajkowska, 2018).

Some evidence suggests that presenting with anxiety symptoms temporarily precedes the development of a depressive disorder (*cf. temporal hypothesis* of emotional disorders). For instance, findings from a recent large scale meta-analytic provide support for the notion that anxiety disorders have, on average, an earlier age of onset than depressive disorders (Solmi et al., 2022). Furthermore, the frequency of patients presenting with depression symptoms without also presenting with anxiety symptoms is estimated to be 5% (Sauer-Zavala and Barlow, 2021). Finally, although depression and anxiety are both negatively associated with positive emotionality, this relationship is stronger in depression (Khazanov and Ruscio, 2016). Taken together, understanding the nuanced differences between depression and anxiety consideration of various factors, including their heterogeneous and multi-layered nature, adaptive functions and their relation with regulatory processes, positive emotionality, motivation, and complex cognitive processes (Eysenck and Fajkowska, 2018).

The common feature of all depressive disorders is the presence of sad, empty, or irritable mood, accompanied by related changes that significantly impair functioning (American Psychiatric Association, 2022). Depression is primarily characterized by two essential features that persist for at least 2 weeks: (1) depressed mood and/or (2) loss of interest or pleasure (i.e., anhedonia) in almost all activities, experienced for the majority of each day (American Psychiatric Association, 2022). It is important to differentiate major depression from bereavement, as sadness induced by bereavement or loss is all too often conflated with major depression (Wakefield et al., 2017), a diagnostic challenge persistently ignored by the DSM classification system. Interestingly, sadness usually decreases as depression shifts towards apathy. However, the frequent overlap between depression and sadness in the early stages of depressive episodes can potentially lead to misconceptions among clients, healthcare providers, and researchers (Watt and Panksepp, 2009; Watt, 2023).

In contrast to depression, anxiety disorders all feature hindering anticipatory thoughts about future threats, nervousness, and uncontrollable worrying (American Psychiatric Association, 2022). Both depression and anxiety involve biased cognitive and emotional processing and high intolerance of uncertainty, which contributes to the high rates of comorbidity observed in these disorders (Beck, 1976; Mathews and MacLeod, 2005; Jensen et al., 2016; McEvoy et al., 2019). However, this poses challenges for diagnosis and treatment, highlighting the need for reliable measurements that are simultaneously responsive to change and prevent non-response bias (Staples et al., 2019).

Brief measures of depression and anxiety can potentially increase the response rates to questionnaires due to their brevity and ease of administration (Kroenke et al., 2003, 2009; Plummer et al., 2016). The Patient Health Questionnaire 2-item scale (PHQ-2) is a streamlined screening tool for depression. It is a shortened version of the more comprehensive Patient Health Questionnaire 9-item scale (PHQ-9; Kroenke et al., 2001), specifically derived from its first two items. The PHQ-2 focuses on assessing the two essential features of depression: depressed mood or hopelessness, and loss of interest or pleasure in almost all activities (Kroenke et al., 2003; Staples et al., 2019; Levis et al., 2020). Similarly, the Generalized Anxiety Disorder 2-item scale (GAD-2) is a concise screening tool for anxiety, derived from the first two items of the more comprehensive Generalized Anxiety Disorder 7-item scale (GAD-7; Spitzer et al., 2006). The GAD-2 focuses on assessing the core features of anxiety disorders: feelings of nervousness and anxiousness, and uncontrollable worrying (Kroenke et al., 2007; Plummer et al., 2016; Staples et al., 2019). The core features of depression and anxiety have previously been assessed reliably with brief self-report instruments (Staples et al., 2019; Byrd-Bredbenner et al., 2021). This study aims corroborate previous findings by assessing the psychometric characteristics, discriminative accuracy, and sensitivity to change of the PHQ-2 and GAD-2 within a Swedish clinical population.

From the preceding discussion, we put forth the following hypotheses. First, we hypothesize that the diagnostic accuracy and internal consistency of brief measures of depression and anxiety will be on par with their full version counterparts. Second, we anticipate a monotonic relationship between higher item scores on the PHQ-2 and GAD-2 and an increased probability of severe depressive and anxiety symptoms.

## 2 Method

### 2.1 Participants and recruitment

Data were obtained as part of a study of internet-delivered, transdiagnostic treatments for anxiety and depression (ClinicalTrials.gov identifier: NCT05016843), conducted in Sweden. Participants were recruited online through a website outlining the study’s aims and constituent parts (Vlaescu et al., 2016). The study was advertised on Facebook but also spread through word of mouth. Thus, the sample consisted of treatment-seeking individuals that became aware of the study through their social circle or social media.

#### 2.1.1 Eligibility criteria

Eligibility criteria were assessed during the study’s screening phase. Participants were required to: (a) be at least 18 years of age; (b) read and write in Swedish; (c) have an internet connection via their mobile phone or computer; and (d) experience at least mild anxiety symptoms (i.e., GAD-7 ≥ 5 points) or mild to moderate depression symptoms (i.e., PHQ-9 ≥ 10 points), or both. Participants were excluded if they: (a) were currently seeking other psychological treatment; (b) had begun or adjusted psychopharmacological treatment for anxiety, worry, or depression within the nearest month from screening; or (c) had severe depression (i.e., PHQ-9 ≥ 20 points) or suicidality (i.e., PHQ-9, item nine score > 2 points) indicated during screening.

### 2.2 Measures and design

Demographic variables and anxiety and depression measurements were collected during screening, followed by weekly measurements of anxiety and depression, and again during post-treatment. Thus, this study employed a cross-sectional study design to evaluate the psychometric properties of the PHQ-2 and GAD-2, brief instruments designed to screen for depression and anxiety, respectively. These scales were chosen due to their brevity and exclusive inclusion of the core characteristics of these disorders. For instance, the PHQ-2 assesses depressed mood and anhedonia (Kroenke et al., 2001) which are necessary, albeit not sufficient, for a diagnosis of depression (American Psychiatric Association, 2022). Similarly, the GAD-2 assesses anxiousness and the uncontrollability of worry (Spitzer et al., 2006), which are common characteristics across all anxiety disorders (American Psychiatric Association, 2022). Thus, these scales capture the core characteristic psychopathology of depression and anxiety, making them highly relevant for monitoring in-treatment fluctuations in the symptomatology of these disorders (*cf.* Fried et al., 2022). Moreover, most anxiety and depressive symptom severity scales do not fully encapsulate the full breadth of the idiosyncrasies of these disorders (Veal et al., 2024), calling into question the need to burden clients and research participants with extensive measures if similar outcomes can be obtained using short-form measures to monitor in-treatment outcomes (McPherson and Armstrong, 2022).

#### 2.2.1 Demographics

Demographic variables gathered during screening included age, gender, socioeconomic status, marital status, household composition, level of education, employment status, mental health characteristics, and prior psychopharmaceutical medication usage.

#### 2.2.2 Patient health questionnaire-9 item and 2-item (PHQ-2)

The Patient health questionnaire-9 (PHQ-9) is a nine-item self-report questionnaire that quantifies the symptom severity of depression (Kroenke et al., 2001). Each item is rated on a 4-point Likert scale ranging from 0 (i.e., “not at all”) to 3 (i.e., “nearly every day”), wherein higher scores indicate greater depressive symptom severity. Total scores range from 0 to 27, where a score of 10 or higher is a diagnostic indicator of depression (Kroenke et al., 2001, 2010). The PHQ-2 comprises the first two items of the PHQ-9 which assess the core features of depression (i.e., depressed mood and anhedonia; Kroenke et al., 2003). These items are (1) little interest or pleasure in doing things, and (2) feeling down, depressed, or hopeless. Total scores range from 0 to 6, where a score of 3 or higher is a diagnostic indicator of depression (Kroenke et al., 2003; Staples et al., 2019; Levis et al., 2020). Prior studies suggest that the PHQ-9 and PHQ-2 possess good accuracy and discrimination ability for screening depressive symptom severity when administered via the internet (Staples et al., 2019; Martin-Key et al., 2022).

#### 2.2.3 Generalized anxiety disorder scale 7-item (GAD-7) and 2-item (GAD-2)

The GAD-7 is a self-report questionnaire that quantifies the symptom severity of generalized anxiety, panic, social anxiety, and post-traumatic stress disorder (Spitzer et al., 2006; Kroenke et al., 2010). Each item is rated on a 4-point Likert scale ranging from 0 (i.e., “not at all”) to 3 (i.e., “nearly every day”), wherein higher scores indicate greater anxiety symptom severity. Total scores range from 0 to 21, where a score of 8 or higher is a diagnostic indicator for the presence of an anxiety disorder (Spitzer et al., 2006; Luo et al., 2019). The GAD-2 comprises the first two items of the GAD-7 which assess the core features of anxiety disorders (Kroenke et al., 2007; Plummer et al., 2016). These items are (1) feeling nervous, anxious or on edge, and (2) not being able to stop or control worrying. Total scores range from 0 to 6, where a score of 3 or higher is a diagnostic indicator of an anxiety disorder with clinical relevance (Kroenke et al., 2007; Plummer et al., 2016; Staples et al., 2019). Prior studies suggest the GAD-7 and GAD-2 possess good accuracy and discrimination ability for screening anxiety severity when administered via the internet (Staples et al., 2019; Martin-Key et al., 2022).

### 2.3 Treatment interventions

Data was collected as part of an ongoing clinical trial comparing cognitive-behavioral therapy (Unified Protocol; Barlow et al., 2017) with psychodynamic Affect Phobia therapy (Julien and O’Connor, 2017). The trial comprised three factors: (a) type of internet-based treatment intervention; (b) treatment length; and (c) effects of access to a clinician-moderated discussion forum. Participants were randomly assigned via a factorial assignment mechanism to one of 12 conditions: Unified Protocol, Affect Phobia, or a waitlist, each for either 8 or 16 weeks, and each with or without access to a clinician-moderated forum.

### 2.4 Statistical analyses

A receiver operating characteristic (ROC) curve analysis was conducted to assess the diagnostic accuracy of the brief measures of depression and anxiety (Hajian-Tilaki, 2013). ROC curve analysis is a quantitative method for combining sensitivity and specificity into a single metric. Defining depression as a score on the PHQ-9 of 10 or more, a variable coded 0 for scores not indicative of depression and 1 for scores indicative of depression was constructed. Similarly, when defining the presence of an anxiety disorder as a score on the GAD-7 of 8 or more, a variable can be coded 0 for scores not indicative of anxiety disorder and 1 for scores indicative of an anxiety disorder. Thereafter, these binary variables were used as outcome variables in a ROC curve analysis to assess the diagnostic accuracy of the PHQ-2 and GAD-2, respectively. Finally, a ROC test was conducted to assess whether the diagnostic accuracy of the PHQ-2 and GAD-2 increased or decreased between pre-treatment screening, weekly treatment measurements, and post-treatment (DeLong et al., 1988; Hajian-Tilaki, 2013).

An item-option characteristic curve analysis was performed for each item in the PHQ-2 and GAD-2. Specifically, so-called expected item score (EIS) plots and item response function (IRF) trace plots were generated using the KernSmoothIRT (Mazza et al., 2014) and mirt (Chalmers, 2012) packages in R. EIS plots display the relationship between individual item scores and their corresponding total scores. As such, EIS plots act as visual tools that facilitate an assessment of the degree to which item scores are monotonically associated with total scores. In contrast, IRF plots display the relationship between different response options within an item and the corresponding probability of manifesting the latent trait under assessment. This method of evaluating the psychometric properties of clinical self-report measurements has been previously used to validate the Beck Depression Inventory–II (BDI-II; Beck et al., 1996).

## 3 Results

### 3.1 Sample characteristics

Descriptive statistics stratified by assignment into an active treatment or waitlist control condition are presented in Table 1.

**Table 1:** Descriptive statistics and comparison of demographics by treatment group randomization.^1^

	Waitlist	Active treatment	Excluded	*p*-value: overall^2^	*p*-value: waitlist vs. active treatment	*p*-value: waitlist vs. excluded	*p*-value: active treatment vs. excluded
	*n* = 826	*n* = 1,651	*n* = 934				
**Education**				<0.001	0.231	<0.001	<0.001
Elementary school	3.3% [2.2, 4.7%]	3.9% [3.1, 5.00%]	9.2% [7.4, 11.2%]				
High school	24.9% [22.0, 28.0%]	28.3% [26.1, 30.5%]	34.3% [31.2, 37.5%]				
College level education <3 years	26.8% [23.8, 29.9%]	25.7% [23.6, 27.9%]	23.7% [21.0, 26.6%]				
College level education >3 years	45.0% [41.6, 48.5%]	42.0% [39.6, 44.5%]	32.8% [29.8, 35.9%]				
**Usage of pharmaceuticals for depression or anxiety**				<0.001	0.154	<0.001	<0.001
No	71.8% [68.6, 74.8%]	74.6% [72.4, 76.6%]	59.0% [55.7, 62.2%]				
Yes	28.2% [25.2, 31.4%]	25.4% [23.4, 27.6%]	41.0% [37.8, 44.3%]				
**Age**	43.0 [42.2, 43.9]	42.6 [42.0, 43.2]	41.1 [40.2, 42.0]	0.002	0.708	0.004	0.011
**Gender**				0.038	0.098	0.022	0.434
Female	85.8% [83.3, 88.1%]	82.1% [80.2, 84.0%]	80.2% [77.5, 82.7%]				
Male	13.6% [11.3, 16.1%]	17.1% [15.4, 19.0%]	18.8% [16.4, 21.5%]				
Other gender identity	0.6% [0.2, 1.4%]	0.7% [0.4, 1.3%]	1.0% [0.4, 1.8%]				
**Living with children under 18**				0.044	0.222	0.222	0.073
No	60.4% [57.0, 63.8%]	56.7% [54.3, 59.1%]	61.6% [58.4, 64.7%]				
Yes	38.1% [34.8, 41.5%]	41.3% [38.9, 43.7%]	35.9% [32.8, 39.1%]				
Complicated	1.45% [0.75, 2.52%]	2.00% [1.38, 2.80%]	2.49% [1.58, 3.71%]				
**Marital status**				<0.001	0.821	<0.001	<0.001
Single/Live alone	33.5% [30.3, 36.9%]	32.3% [30.0, 34.6%]	41.9% [38.7, 45.1%]				
Live alone but in a relationship	10.7% [8.63, 13.0%]	10.8% [9.33, 12.4%]	12.7% [10.6, 15.0%]				
Married/Live with partner	55.8% [52.3, 59.2%]	56.9% [54.5, 59.3%]	45.5% [42.2, 48.7%]				
**Occupation**				<0.001	0.512	<0.001	<0.001
Working	69.0% [65.7, 72.1%]	68.2% [65.9, 70.4%]	57.5% [54.2, 60.7%]				
Studying	11.9% [9.74, 14.3%]	13.6% [12.0, 15.4%]	15.4% [13.1, 17.9%]				
Seeking work	7.0% [5.4, 9.0%]	5.9% [4.8, 7.1%]	8.6% [6.8, 10.5%]				
Retired	4.2% [3.0, 5.8%]	4.7% [3.8, 5.9%]	4.7% [3.4, 6.2%]				
Parental leave	1.1% [0.5, 2.1%]	1.6% [1.0, 2.3%]	0.9% [0.4, 1.7%]				
Sick leave	6.8% [5.2, 8.7%]	6.0% [4.9, 7.3%]	13.1% [11.0, 15.4%]				
**My socioeconomic status is…**				<0.001	0.922	<0.001	<0.001
Much worse than other people’s	5.00% [3.6, 6.7%]	5.3% [4.3, 6.5%]	11.7% [9.7, 13.9%]				
Worse than other people’s	23.5% [20.6, 26.5%]	22.8% [20.8, 24.9%]	24.6% [21.8, 27.5%]				
About the same as other people’s	43.1% [39.7, 46.6%]	44.6% [42.2, 47.1%]	41.1% [37.9, 44.4%]				
Better than other people’s	25.5% [22.6, 28.7%]	24.7% [22.6, 26.8%]	19.9% [17.4, 22.6%]				
Much better than other people’s	2.9% [1.9, 4.3%]	2.6% [1.9, 3.5%]	2.7% [1.8, 4.0%]				
**Above clinical cut-offs**							
**Screening**							
PHQ-9 ≥ 10	70.2% [67.1, 73.3%]	69.7% [67.5, 71.9%]	69.0% [66.1, 72.0%]	0.888	0.964	0.856	0.933
PHQ-2 ≥ 3	56.8% [53.4, 60.2%]	57.9% [55.5, 60.3%]	68.6% [65.6, 71.6%]	< 0.001	0.85	< 0.001	< 0.001
GAD-7 ≥ 8	64.3% [61.0, 67.6%]	66.1% [63.9, 68.4%]	64.3% [61.2, 67.4%]	0.525	0.631	> 0.999	0.610
GAD-2 ≥ 3	59.6% [56.2, 62.9%]	63.8% [61.5, 66.2%]	61.9% [58.8, 65.0%]	0.113	0.096	0.571	0.595
**Post-assessment** ^**3**^							
PHQ-9 ≥ 10	34.1% [29.6, 38.9%]	31.4% [26.2, 37.0%]			0.497		
PHQ-2 ≥ 3	23.8% [19.8, 28.2%]	24.7% [19.9, 30.0%]			0.860		
GAD-7 ≥ 8	35.6% [31.0, 40.4%]	32.5% [27.2, 38.2%]			0.446		
GAD-2 ≥ 3	35.6% [31.0, 40.4%]	32.5% [27.2, 38.2%]			0.446		

^1^Numbers reflect percentages in all cases except for age which is wherein the mean is reported. ^2^Chi-square significance tests were used for group comparisons. ^3^Represents comparisons made between participants included in the study (i.e., screened into the study).

### 3.2 Discriminative validity

A ROC curve analysis was conducted on the short-form questionaries for pre-treatment screening, weekly treatment measurements, and post-assessment scores (see Table 2). During pre-treatment screening, the PHQ-2 had highly acceptable discriminative validity (AUC between 0.780 and 0.809), and the GAD-2 had excellent discriminative validity (AUC between 0.858 and 0.882). This suggests that brief measures of depression and anxiety can readily detect treatment-seeking individuals with scores that indicate an anxiety or depressive disorder before the onset of a psychotherapeutic treatment intervention.

**Table 2:** Area under the curve at baseline, post-assessment, and scores throughout the trial indicative of diagnosis of depression or anxiety.^1^

	Area under the curve (95% CI)	
	PHQ-2	GAD-2
**Pre-treatment screening**	0.795 (0.780–0.809)	0.870 (0.858–0.882)
**During treatment** ^2^	0.803 (0.797–0.809)	0.886 (0.881–0.890)
**Post-assessment**	0.802 (0.797–0.808)	0.883 (0.878–0.888)

^1^AUC: acceptable = 0.70–0.79; excellent = ≥ 0.80. Calculated with the criterion of scores ≥ 3 for both measures. ^2^Computed from data collected throughout the duration of treatment.

Similarly, for weekly anxiety and depressive symptom severity indices collected during the course of active treatment, discriminative validity was excellent for both the PHQ-2 (AUC between 0.797 and 0.809) and GAD-2 (AUC between 0.881 and 0.890). This suggests that brief measures of depression and anxiety have high discriminative ability in distinguishing between individuals that may and may not qualify for a diagnosis of depression and anxiety during active psychotherapy. Finally, the discriminative validity was excellent at post-assessment for both the PHQ-2 (AUC between 0.797 and 0.808) and GAD-2 (AUC between 0.878 and 0.888), in turn, suggesting that the brief measures of depression and anxiety reliably detect individuals that may qualify for a diagnosis of depression and anxiety after a psychotherapy has been provided.

The difference in AUC between pre-treatment screening and post-assessment was significant for the GAD-2 [*D* = −1.98, *p* = 0.049], indicating that the GAD-2 had greater discriminative ability for anxiety at post-assessment than during pre-treatment screening. However, the difference in AUC between pre-treatment screening and post-assessment was nonsignificant for the PHQ-2 [*D* = −0.95, *p* = 0.34], indicating no change in the discriminative ability in the brief measure of depression between pre-treatment screening and post-assessment. Sensitivity and specificity of the scores on the PHQ-2 and GAD-2 collected during pre-treatment are presented in Table 3.

**Table 3:** Pre-treatment sensitivity and specificity of the PHQ-2 and GAD-2.^1^

	Sensitivity	Specificity	Likelihood ratio	Youden’s *J*
**PHQ-2**				
≥1	0.93	0.72	3.37	0.65
≥2	0.84	0.76	3.54	0.60
≥3	0.62	0.90	6.34	0.52
≥4	0.50	0.96	12.21	0.46
≥5	0.42	0.99	30.31	0.40
≥6	0.37	0.99	55.71	0.36
**GAD-2**				
≥1	0.98	0.67	2.99	0.65
≥2	0.96	0.74	3.64	0.69
≥3	0.79	0.92	10.12	0.72
≥4	0.63	0.98	32.29	0.61
≥5	0.50	1.00	NA	0.50
≥6	0.43	1.00	NA	0.43

^1^Positive likelihood ratio = sensitivity/(1-specificity). Youden’s *J* statistic = sensitivity + specificity – 1. Values range between 0 (low diagnostic accuracy) and 1 (high diagnostic accuracy). NA, not applicable.

The difference in AUC between pre-treatment screening data and weekly treatment data was significant for the GAD-2 [*D* = −2.35, *p* = 0.002], indicating that the GAD-2 had greater discriminative ability for anxiety in the weekly treatment measurements than during pre-treatment screening. However, the difference in AUC between pre-treatment screening data and weekly treatment data was nonsignificant for the PHQ-2 [*D* = −1.05, *p* = 0.30], indicating no change in the discriminative ability in the brief measure of depression between pre-treatment screening and weekly treatment measurements. Finally, the difference in AUC between the weekly treatment and post-assessment measurements was nonsignificant for both the GAD-2 [*D* = 0.72, *p* = 0.47] and the PHQ-2 [*D* = 0.18, *p* = 0.86], indicating no changes in the discriminative ability for the brief measures of anxiety and depression between weekly treatment measurements and post-assessment.

Taken together, brief measures such as the PHQ-2 and GAD-7 can effectively distinguish individuals who may be eligible for a diagnosis of depression and anxiety, not only prior to but also during and following the completion of an active treatment intervention. Furthermore, the discriminative ability of the GAD-2 was greater during active treatment and at post-assessment than during pre-treatment screening, although no such differences were found for the PHQ-2. This greater discriminative ability in the GAD-2 is likely a result of the pre-treatment screening dataset consisting both of participants that were included and excluded from the study.

Sensitivity and specificity for the PHQ-2 and GAD-2 during treatment and at post-assessment are reported in Table 4.

**Table 4:** Sensitivity and specificity of the PHQ-2 and GAD-2 throughout the duration of treatment and at post-assessment.^1^

	Sensitivity	Specificity	Likelihood ratio	Youden’s *J*
**During treatment**
PHQ-2 ≥ 3	0.83	0.84	5.05	0.67
GAD-2 ≥ 3	0.90	0.89	7.83	0.78
**Post-assessment**
PHQ-2 ≥ 3	0.11	1.00	NA	0.11
GAD-2 ≥ 3	0.93	0.20	1.16	0.13

^1^Positive likelihood ratio = sensitivity/(1-specificity). Youden’s J statistic = sensitivity + specificity – 1. Values range between 0 (low diagnostic accuracy) and 1 (high diagnostic accuracy). NA, not applicable.

### 3.3 Other psychometric properties

The internal consistency of the brief measures of depression and anxiety was comparable to the long-format versions and between different instances of data collection (see Table 5).

**Table 5:** Internal validity of the brief measures of anxiety and depression.^1^

		95% CI	
	Standardized Cronbach’s alpha	Lower	Upper
**Pre-treatment screening** ^**2**^			
PHQ-9	0.81	0.80	0.82
PHQ-2	0.76	0.74	0.78
GAD-7	0.84	0.83	0.85
GAD-2	0.80	0.78	0.81
**During treatment** ^**3**^			
PHQ-9	0.85	0.84	0.85
PHQ-2	0.82	0.81	0.82
GAD-7	0.87	0.86	0.87
GAD-2	0.82	0.81	0.83
**Post-assessment** ^**4**^			
PHQ-9	0.87	0.86	0.89
PHQ-2	0.86	0.83	0.89
GAD-7	0.87	0.86	0.89
GAD-2	0.83	0.81	0.86

^1^Missing observations were omitted in computations. ^2^3,401 observations in both the PHQ and GAD (no observation missing). ^3^Computed from data collected throughout treatment with 18,767 observations in the PHQ (55,543 observations missing) and 18,696 in the GAD (55,614 observations missing). ^4^712 observations in the PHQ (1765 observations missing) and 705 observations in the GAD (1772 observations missing).

### 3.4 Item-option characteristic curves

**Figure 1:**
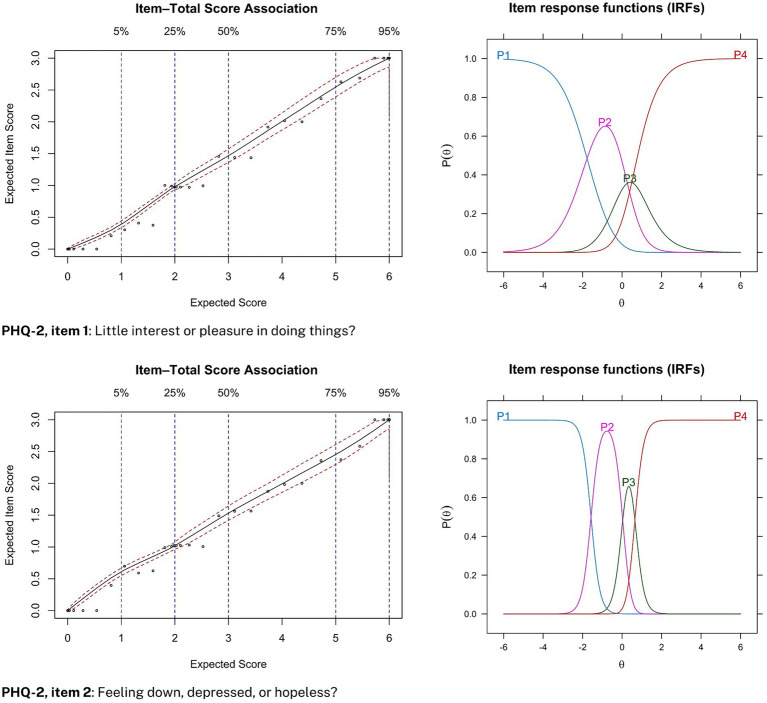
Item-option characteristic curves for the PHQ-2 during screening. Expected item score (EIS) plots are displayed on the left side and item response functions (IRFs) are displayed on the right side.

**Figure 2:**
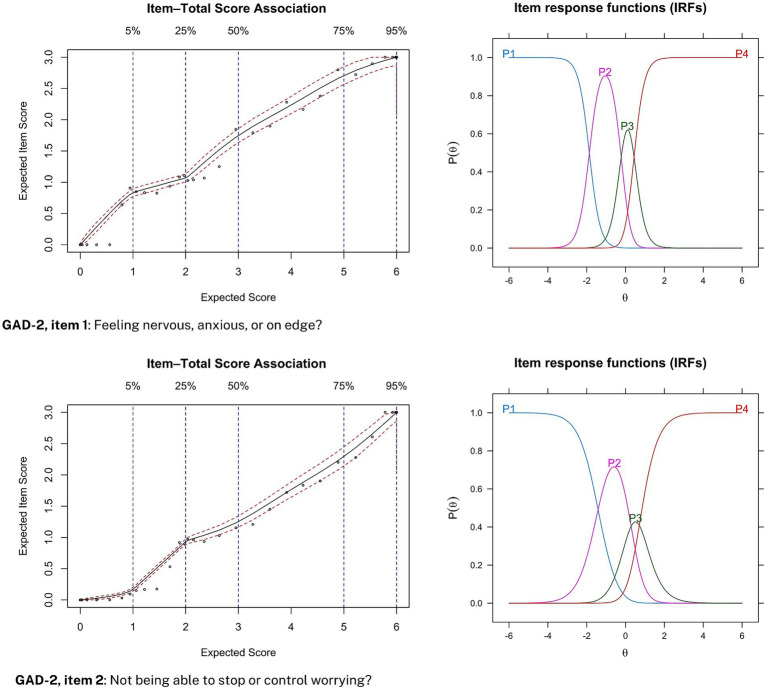
Item-option characteristic curves for the GAD-2. Expected item score (EIS) plots are displayed on the left side and item response functions (IRFs) are displayed on the right side.

Item-option characteristic curves are plotted for each item in the PHQ-2 and GAD-2 during pre-treatment screening (see Figures 1, 2). Specifically, expected item scores (EIS) and their corresponding total scores are plotted on the left sides of Figures 1, 2, and item response functions (IRFs) are plotted on the right sides. EIS plots facilitate aid the assessment of monotonic relationships between item scores and total scores, while IRFs display how different response options within items are predictive of the latent trait captured by the questionnaire.

For depressive symptom severity, increasing item category endorsement was monotonically associated with an increased total score for both items in the PHQ-2 (see Figure 1, left side). Moreover, endorsing the least severe response category was consistently predictive of a low probability of suffering from depression, while an endorsement of the most severe response category (i.e., 3-points, “nearly every day”) was consistently predictive of a high probability of latent depressive disorder (see Figure 1, right side). Similar item-option characteristic curves were obtained for data collected during weekly treatment measurements.

Similarly, for anxiety symptom severity, increasing item category endorsement was monotonically associated with an increased total score for both items in the GAD-2 (see Figure 2, left side). Moreover, endorsing the least severe response category was consistently predictive of a low probability of suffering from anxiety, while an endorsement of the most severe response category (i.e., 3-points, “nearly every day”) was consistently predictive of a high probability of a latent anxiety disorder (see Figure 2, right side). Similar item-option characteristic curves were obtained for data collected during weekly treatment measurements.

## 4 Discussion

The PHQ-2 and GAD-2 are very brief measures of depression and anxiety. This study aimed to assess their discriminative accuracy, psychometric properties, and responsiveness to change. The results partly support our hypothesis about comparable diagnostic accuracy and internal consistency between the brief measures and their full version counterparts. Both the PHQ-2 and GAD-2 showed excellent discriminative validity during the trial. Moreover, both brief measures showed comparable internal stability during pre-screening, post-assessment, and the trial. During pre-treatment screening, the PHQ-2 showed acceptable discrimination and the GAD-2 showed excellent discriminative ability. These results mirror previous studies (see, e.g., Staples et al., 2019), where both instruments show excellent or acceptable discriminative validity, respectively, at baseline and follow-up measurements.

The results also support our hypothesized positive monotonic relationship between individual item scores and probability of depressive and anxiety symptom severity. For both the PHQ-2 and GAD-2, an endorsement of the most severe response option (i.e., 3-points) was associated with a high probability of having latent depressive and anxiety disorder, respectively. Put differently, participant rank-ordered magnitudes of latent-trait depression and anxiety was preserved between items 1 and 2 on both the PHQ-2 and GAD-2. This statistical relationship corroborates guidelines for the PHQ-2 (Kroenke et al., 2003; Levis et al., 2020) and GAD-2 (Kroenke et al., 2007; Plummer et al., 2016), which suggest that a total score of 3 or greater is clinically relevant. This also dovetails with our finding that a cut-off score of 3 provides optimal sensitivity and specificity for both the PHQ-2 and GAD-2. However, some evidence does suggest that a cut-off score for the PHQ-2 should be a score of 2 or greater when used in practice. For instance, a recent meta-analysis found that combining PHQ-2 (with cut-off ≥2) and PHQ-9 (with cut-off ≥10) yielded similar sensitivity estimates with higher specificity than only PHQ-9 with cut-off scores of 10 or greater (Levis et al., 2020). However, this trade-off drastically lowers specificity if the PHQ-2 is not followed up on with the PHQ-9 (Staples et al., 2019).

Taken together, the present study partly replicates previous studies that suggest the PHQ-2 and GAD-2 to be viable options to detect depression and anxiety symptom severity, even when they are administered via the internet (Kroenke et al., 2003; Plummer et al., 2016; Staples et al., 2019; Levis et al., 2020; Byrd-Bredbenner et al., 2021). Our results suggest that the PHQ-2 and GAD-2 are excellently suited for pre-treatment screening, monitoring individuals over the course of treatment, and at post-assessment. As such, our analyses provide robust support for the use of brief measures to monitor treatment outcomes, although some nuanced information may be lost for individual participants. Therefore, we recommend that these brief measures be chiefly used to monitor treatment outcomes during treatment (e.g., using ecological momentary experience sampling), as they do provide valuable symptomatology insights without unnecessarily burdening respondents. Specifically, our results provide support for the usage of brief measures to monitor in-treatment fluctuations in depressive and anxiety symptom severity using ecological momentary experience sampling protocols.

Due to their brevity, these brief measures can be administered with greater frequency than their full-scale counterparts, thereby potentially informing mechanisms of change in treatment studies. Furthermore, although more frequent measurements might initially seem burdensome for participants, research on habit formation indicates that higher frequency of a behavior enhances its automaticity (Gardner et al., 2012; McCloskey and Johnson, 2019). Thus, incorporating these brief measures into ecological momentary experience sampling protocols could counterintuitively increase response rates to self-report questionnaires that monitor in-treatment fluctuations in psychopathological symptoms.

This study has several limitations, the most significant of which being the lack of clinical diagnostic interviews. While such interviews are the gold standard for confirming mental disorder diagnoses such as depression and anxiety (Carlbring et al., 2002), we used summation scores on the PHQ-9 and GAD-7. Previous research has shown these to be reliable indicators of depression and anxiety, thus allowing us to categorize people as either with or without these disorders. This method, however, does limit the present analysis. Nonetheless, our approach is supported by prior findings indicating that the cutoff points on both the PHQ-9 (Kroenke et al., 2010; Martin-Key et al., 2022) and the GAD-7 (Johnson et al., 2019; Byrd-Bredbenner et al., 2021; Martin-Key et al., 2022) routinely emerge as valid indicators of depressive and anxiety disorders. Furthermore, only treatment-seeking participants with scores indicative of anxiety and/or depressive symptoms were included in the study. Another notable limitation is a considerable amount of missing data and few participants with data available at the follow-up measurement. Only 712 individuals provided data at follow-up compared to 3,401 at pre-treatment screening. As such, greater uncertainty is to be expected in the follow-up post-assessment. Finally, the study may be somewhat limited by a homogenous treatment-seeking sample. However, measures of psychopathology are primarily intended to be administered to a treatment-seeking population, and thus it is not self-evident that the sample characteristics limit the generalizability of the present findings; rather the sample can be conceived of as representative of a treatment-seeking population which is precisely the population that is pertinent for analyses of this nature. Despite the abovementioned limitations, the study found that brief measures of depression and anxiety are viable in a Swedish setting and revealed a strong monotonic relationship between item scores and total scores, corroborated by a large sample size during pre-treatment which, in turn, increases certainty and stability in item parameter assessments.

The present study has numerous strengths. Chiefly, by leveraging data from participants that were included and excluded from the study in our psychometric properties analysis in the pre-treatment screening data, this study does not suffer from a restriction of range for the pre-treatment screening data. Other strengths include the exclusive inclusion of treatment-seeking individuals in the study and large sample sizes in pre-treatment and treatment data. Finally, this study further corroborates previous findings that have suggested brief measures of anxiety and depressive disorders to be viable alternatives by replicating their findings in a Swedish context (e.g., Staples et al., 2019).

### 4.1 Future directions

Brief measures of depression and anxiety can potentially increase the response rates to questionnaires due to their brevity and ease of administration (Kroenke et al., 2003; Plummer et al., 2016). Future studies could incorporate them in ecological momentary experience sampling protocols (Verhagen et al., 2022), with frequent collection of indices of anxiety and depression, thereby increasing the representation of data from individuals with varying levels of mental disorder symptoms. Moreover, such studies should evaluate whether response rates, on average, increase as a result of more frequent measurement instances. Finally, this study did not conduct a proper item response theory analysis, but rather evaluates monotonicity for the PHQ-2 and GAD-2 and provides a preliminary analysis of the item response functions. Future studies could further analyze these brief measures using a nonparametric item response theory modelling (i.e., Mokken scale analysis; Sijtsma and Molenaar, 2002) to further validate the PHQ-2 and GAD-2.

## Data availability statement

The raw data supporting the conclusions of this article will be made available by the authors, without undue reservation.

## Ethics statement

The studies involving humans were approved by The Swedish Ethical Review Authority (Dnr 2021-00034). The studies were conducted in accordance with the local legislation and institutional requirements. The participants provided their written informed consent to participate in this study.

## Author contributions

JH: Conceptualization, Methodology, Writing – review & editing, Data curation, Formal analysis, Investigation, Visualization, Writing – original draft. PC: Conceptualization, Methodology, Writing – review & editing, Project administration, Resources, Supervision.
